# A Mammal-Specific *Doublesex* Homolog Associates with Male Sex Chromatin and Is Required for Male Meiosis

**DOI:** 10.1371/journal.pgen.0030062

**Published:** 2007-04-20

**Authors:** Shinseog Kim, Satoshi H Namekawa, Lisa M Niswander, Jeremy O Ward, Jeannie T Lee, Vivian J Bardwell, David Zarkower

**Affiliations:** 1 Department of Genetics, Cell Biology, and Development, University of Minnesota, Minneapolis, Minnesota, United States of America; 2 Biochemistry, Molecular Biology, and Biophysics Graduate Program, University of Minnesota, Minneapolis, Minnesota, United States of America; 3 Department of Molecular Biology, Massachusetts General Hospital, Harvard Medical School, Boston, Massachusetts, United States of America; 4 Department of Genetics, Harvard Medical School, Boston, Massachusetts, United States of America; 5 Howard Hughes Medical Institute, Harvard Medical School, Boston, Massachusetts, United States of America; 6 Department of Biology, Middlebury College, Middlebury, Vermont, United States of America; Cornell University, United States of America

## Abstract

Gametogenesis is a sexually dimorphic process requiring profound differences in germ cell differentiation between the sexes. In mammals, the presence of heteromorphic sex chromosomes in males creates additional sex-specific challenges, including incomplete X and Y pairing during meiotic prophase. This triggers formation of a heterochromatin domain, the XY body. The XY body disassembles after prophase, but specialized sex chromatin persists, with further modification, through meiosis. Here, we investigate the function of DMRT7, a mammal-specific protein related to the invertebrate sexual regulators Doublesex and MAB-3. We find that DMRT7 preferentially localizes to the XY body in the pachytene stage of meiotic prophase and is required for male meiosis. In *Dmrt7* mutants, meiotic pairing and recombination appear normal, and a transcriptionally silenced XY body with appropriate chromatin marks is formed, but most germ cells undergo apoptosis during pachynema. A minority of mutant cells can progress to diplonema, but many of these escaping cells have abnormal sex chromatin lacking histone H3K9 di- and trimethylation and heterochromatin protein 1β accumulation, modifications that normally occur between pachynema and diplonema. Based on the localization of DMRT7 to the XY body and the sex chromatin defects observed in *Dmrt7* mutants, we conclude that DMRT7 plays a role in the sex chromatin transformation that occurs between pachynema and diplonema. We suggest that DMRT7 may help control the transition from meiotic sex chromosome inactivation to postmeiotic sex chromatin in males. In addition, because it is found in all branches of mammals, but not in other vertebrates, *Dmrt7* may shed light on evolution of meiosis and of sex chromatin.

## Author Summary

Genes related to the sexual regulator *Doublesex* of *Drosophila* have been found to control sexual development in a wide variety of animals, ranging from roundworms to mammals. In this paper, we investigate the function of the *Dmrt7* gene, one of seven related genes in the mouse. Female mammals are XX and males are XY, a chromosomal difference that presents specific challenges during the meiotic phase of male germ cell development. Some of these are thought to be overcome by incorporating the X and Y chromosomes into a specialized structure called the XY body. We find that DMRT7 protein is present in germ cells, localizes to the male XY body during meiosis, and is essential for male but not female fertility. The XY body normally is altered by recruitment of additional proteins and by specific modifications to histone proteins between the pachytene and diplotene stages of meiosis, but modification of the “sex chromatin” in *Dmrt7* mutant cells is abnormal during this period. Because *Dmrt7* is found in all branches of mammals, but not in other vertebrates, these results may indicate some commonality in regulation of sex chromatin among the mammals.

## Introduction

Sexual differentiation generates anatomical, physiological, and behavioral dimorphisms that are essential for sexual reproduction. Many of these dimorphisms affect somatic cells, but the sexual dimorphisms that most directly mediate sexual reproduction are those of the gametes themselves. Gametes differ between the sexes in size and morphology, sometimes dramatically so, reflecting their very different roles in zygote formation. Indeed, the morphology of the gametes is what defines sex: females are the sex that produces the larger gametes and males produce the smaller ones.

Mammalian meiosis is regulated sex-specifically starting in embryogenesis and continuing through much of adult life (reviewed in [1]). For example, the timing and synchrony of meiosis are very different in the two sexes. In females, germ cells synchronously initiate meiosis in the embryo and arrest during meiotic prophase I. After puberty, oocytes are selectively recruited for ovulation, when they proceed to metaphase II and then complete meiosis after fertilization occurs [2]. In contrast, male meiosis occurs entirely postnatally, without the arrest periods found in females. In females, each meiosis can produce a single haploid oocyte (and two extruded polar bodies), whereas each male meiosis can produce four haploid spermatocytes.

Other meiotic processes, such as recombination and chromosome pairing (synapsis), occur in both sexes but operate somewhat differently. For example, there is a higher failure rate for meiosis in females, with human oocyte aneuploidy rates up to 25% versus about 2% in human sperm [3], and this may indicate that the checkpoints controlling and monitoring the events of meiotic progression in males are more stringent. Consistent with this idea, genetic analysis of a number of meiotic regulatory genes in the mouse has demonstrated a much stronger requirement in males than in females [1,4].

The existence of heteromorphic sex chromosomes, such as the XX/XY system of mammals, creates sex-specific challenges. One is the need for mechanisms to balance expression of sex-linked genes between the sexes, which in mammals is accomplished by X chromosome inactivation in females [5,6]. In male germ cells there is another sex-specific consideration during meiosis. In prophase I, when the homologous chromosomes synapse and homologous recombination occurs, X and Y chromosome pairing is limited to a region termed the pseudoautosomal region, leaving large portions of each chromosome unpaired. In eutherian and marsupial mammals, these unpaired chromosome regions are associated with a specialized chromatin domain termed the XY body or sex body. The function of the XY body is uncertain [7–11], but there is evidence that it is essential for male meiotic progression [12].

Several proteins are reported to localize to the XY body, including BRCA1, ATR, the histone variant H3.3, and modified histones such as ubiquitinated H2A (Ub-H2A) and phosphorylated H2AX (γH2AX) [12–15]. In the XY body, the sex chromosomes are transcriptionally silenced in a process termed meiotic sex chromosome inactivation (MSCI). The XY body disappears after pachynema; however, many sex-linked genes remain transcriptionally silent into spermiogenesis [16]. This maintenance of silencing is associated with a distinct set of chromatin marks that define a sex chromatin domain termed postmeiotic sex chromatin (PMSC) [16,17].

Regulators of sexual differentiation have been identified in a number of organisms, but in contrast to many other developmental processes, such as axial patterning or development of many body parts, the molecular mechanisms that regulate sexual differentiation are highly variable between phyla. A notable exception involves genes related to *doublesex (dsx)* of *Drosophila,* which share a Doublesex/MAB-3 DNA-binding motif called the DM domain [18,19]. DM domain–encoding genes have been shown to regulate various aspects of sexual differentiation in insects, nematodes, and mammals [20]. The *mab-3* gene of Caenorhabditis elegans has been shown to function analogously to DSX in several respects and can be functionally replaced by the male isoform of DSX, suggesting that the similarity in the sequence of these genes may stem from conservation of an ancestral DM domain sexual regulator [18,21,22].

Vertebrates also have DM domain genes, and analysis to date, although limited, has shown that these genes also control sexual differentiation. Mammals have seven DM domain genes (*Dmrt* genes), several of which exhibit sexually dimorphic mRNA expression [23,24]. The best studied of these genes, *Dmrt1,* is expressed in the differentiating male genital ridges and adult testis of mammals, birds, fish, and reptiles, and a recently duplicated *Dmrt1* gene functions as the Y-linked testis-determining gene in the Medaka fish [25–29]. Human *DMRT1* maps to an autosomal locus, which, when hemizygous, is associated with defective testicular development and consequent XY feminization [30]. Similarly, mice homozygous for a null mutation in *Dmrt1* have severe defects in testis differentiation involving both germ cells and Sertoli cells [31]. Female mice mutant in *Dmrt4* have polyovular follicles, indicating that this gene also plays a role in gonadal development [32]. It appears from these studies that the involvement of DM domain genes in sexual differentiation is ancient and conserved. However, vertebrate *Dmrt* gene function is not limited to sexual differentiation: *Dmrt2* is required in both sexes for segmentation in mice and fish [33–35].

Here, we have investigated the expression and function of the *Dmrt7* gene in the mouse*. Dmrt7* is expressed only in the gonad, and, unlike the other *Dmrt* genes, appears to be present exclusively in mammals and not in nonmammalian vertebrates [23,36]. We find that DMRT7 protein is expressed only in germ cells and is selectively localized to the XY body of male pachytene germ cells. To test its function, we generated a conditional null mutation of *Dmrt7* in the mouse. We find that *Dmrt7* is required in males for progression beyond the pachytene stage of meiotic prophase but is not required in females. In rare mutant cells that survive to diplonema, we observed sex chromatin abnormalities. Based on these observations, we suggest that *Dmrt7* plays a critical role in a male-specific chromatin transition between pachynema and diplonema during meiotic prophase.

## Results

### Expression of DMRT7 Proteins in Testis

**Figure 1:**
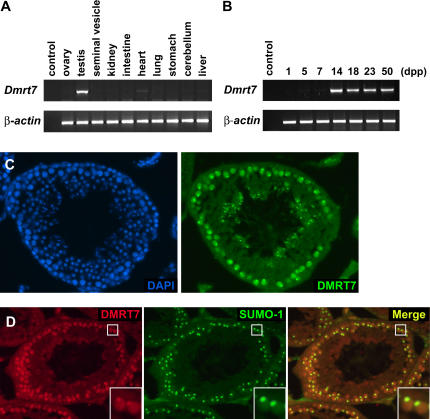
Expression of *Dmrt7* mRNA and Protein (A) RT-PCR analysis of *Dmrt7* mRNA from ten organs of adult mouse. cDNA from each organ was amplified with primers specific for *Dmrt7* (top row) and β-*actin* (bottom row). (B) *Dmrt7* mRNA expression during the first round of spermatogenesis. cDNAs obtained from testis at the indicated days after birth were amplified as in (A). (C) DMRT7 protein expression. Immunofluorescence of testis sections from 6-wk-old male stained with antibody to DMRT7 (green) and DAPI (blue). (D) DMRT7 subcellular localization to XY body. Testis sections from 6-wk-old male stained with antibodies to DMRT7 (red) and SUMO-1 (green). SUMO-1 is localized to the XY body. Right-most panel shows merge of other two panels. Inserts show higher magnification of pachytene spermatocytes with XY bodies.

Our previous mRNA expression analysis suggested a possible meiotic function for *Dmrt7,* based on the expression of *Dmrt7* mRNA in the fetal gonads of the two sexes [23]. In the fetal ovary, *Dmrt7* mRNA was detected primarily from E13.5 to E15.5, the time during which meiosis progresses from pre-meiotic replication to the pachytene stage [4], whereas *Dmrt7* expression in the non-meiotic fetal testis was very low. Because this earlier work did not examine adult *Dmrt7* expression, we first performed reverse transcriptase (RT)-PCR on mRNA from ten adult organs and detected strong *Dmrt7* mRNA expression in the testis and a trace of expression in heart, but not in any other tissue tested (Figure 1A). We examined the timing of *Dmrt7* mRNA expression during postnatal testis development and detected strong expression beginning at 2 wk, which roughly coincides with the onset of the pachytene stage during the first synchronous wave of spermatogenesis (Figure 1B) [37].

To investigate DMRT7 protein expression, we generated an antibody against the C-terminal portion of the protein. The antibody was raised against a unique region lacking the DM domain in order to avoid cross-reaction with other DM domain proteins. Immunofluorescent staining with purified DMRT7 antisera showed that DMRT7 protein is expressed predominantly in mid- to late-pachytene spermatocytes (Figure 1C), as well as in sperm, and is not detectable in other germ cell types including spermatogonia and round spermatids. We did not detect DMRT7 protein in somatic cells such as Sertoli cells, peritubular myoid cells, or Leydig cells. To more precisely determine the pachytene stages of DMRT7 expression, we double-stained with an antibody to GATA1, which is expressed in Sertoli cells from stages VII to IX [38]. This confirmed that DMRT7 is expressed in mid- to late-pachytene spermatocytes, starting slightly earlier than stage VII and extending through stage IX (unpublished data).

Within pachytene spermatocytes, DMRT7 is concentrated in the XY body, or sex body, a densely staining chromatin domain that harbors the sex chromosomes. These undergo transcriptional inactivation and heterochromatinization as a result of their incomplete pairing during prophase of mammalian male meiosis [17]. To verify DMRT7 protein expression in the XY body, we double-stained mouse testis sections for DMRT7 and small ubiquitin-related modifier 1 (SUMO-1), which is concentrated in the XY body during pachynema [39,40]. DMRT7 and SUMO-1 were colocalized, confirming that DMRT7 protein is preferentially localized to the XY body (Figure 1D). We also confirmed XY body localization of DMRT7 by double staining for other markers including Ub-H2A and γH2AX (unpublished data). DMRT7 is not preferentially localized to the XY body at all stages but instead is dynamic. Based on epithelial staging, it appears that DMRT7 localizes to the XY body from mid- to late-pachynema, becomes diffusely distributed in late-pachynema, and disappears in diplonema (unpublished data). This localization was confirmed by staining of meiotic spreads (Figure S1). DMRT7 also is specifically localized in sperm, with antibody staining mainly in the perinuclear ring of the sperm head manchette. This staining coincided with the epithelial stages in which DMRT7 localizes to the XY body in spermatocytes (Figure 1C and 1D).

### Targeted Deletion of *Dmrt7*

To establish the functional requirement for *Dmrt7,* we generated *Dmrt7* ^−/−^ mice by targeted disruption in embryonic stem (ES) cells using a strategy diagrammed in Figure S2A. The *Dmrt7* gene has nine exons with the DM domain encoded by the second and third exons. Because the DM domain is essential for function of other genes, including *mab-3, mab-23,* and *dsx* [18,19,41], we generated a conditionally targeted “floxed” allele in which the DM domain–containing exons of *Dmrt7* are flanked by recognition sites for the Cre recombinase (*loxP* sites). The targeting vector also contained a neomycin resistance cassette *(neo)* flanked by Flpe recognition sites. The removal of these sequences by Cre-mediated recombination eliminates the DM domain and the translational start site, thus generating a putative null allele. We identified three homologously targeted ES cell clones by Southern blotting (Figure S2B) and injected cells from two clones into C57BL/6 blastocysts. Chimeric animals from both cell lines transmitted the targeted allele through the germ line. Targeted animals were bred to β*-actin Cre* mice to delete the DM domain–encoding exons, generating the *Dmrt7^−^* allele, or to *Flpe* transgenic mice to delete the *neo* cassette, generating the *Dmrt7^flox^* allele. *Dmrt7^+/−^* mice were interbred to generate *Dmrt7^−/−^* mice. To confirm the lack of functional DMRT7 protein in *Dmrt7^−/−^*testes, we stained meiotic spreads from *Dmrt7* mutants (Figure S1) and sections from mutant testes (Figure S2C) and carried out western blot analysis (unpublished data). In each case, we detected no DMRT7 protein in the mutant testes.

### *Dmrt7* Is Required for Male but Not Female Gametogenesis

**Figure 2:**
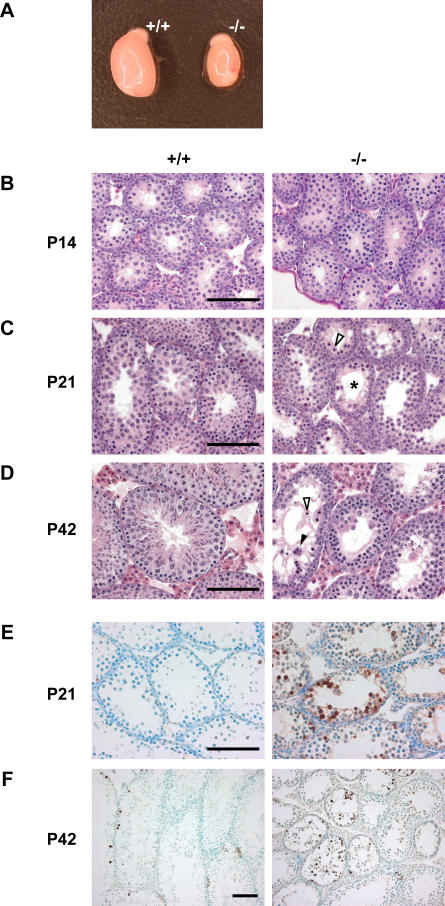
Reduced Testis Size and Germ Cell Apoptosis in Mice with Targeted Deletion in *Dmrt7* (A) Testes from a 6-wk-old wild-type (+/+) mouse and a homozygous (−/−) *Dmrt7* mutant littermate. (B–D) Sections of testes from 14-d-old (B), 21-d-old (C), and 42-d-old mice (D) stained with hematoxylin and eosin. Wild-type is in left column and mutant in right. No significant difference is observed at 14 d (B), but by 21 d some tubules are lacking abundant spermatocytes (C, asterisk) or cells with typical apoptotic morphology are present (open arrowhead, C and D). Mutant tubules contain multinucleate cells (closed arrowhead, D). (E and F) TUNEL labeling of *Dmrt7*-deficient mouse testes. Testes from wild-type and homozygous mutant littermates were analyzed by TUNEL labeling to detect apoptotic cells. Testis sections from 21-d-old (E) and 6-wk-old mice (F). Apoptotic cells (brown) are much more abundant in seminiferous tubules of homozygous *Dmrt7* mutant mice relative to wild-type. Bars in (B–F) represent 100 μm.

Breeding of *Dmrt7* heterozygotes produced homozygous mutant progeny of both sexes at the expected frequency (63/264; 23%). Male and female homozygous mutants were viable, grew to adulthood normally, and exhibited normal sexual behavior. Female homozygotes were fertile, produced litters of normal size, and had no obvious ovarian abnormalities as judged by histological analysis (unpublished data). In contrast, *Dmrt7* homozygous mutant males were completely infertile and had testes about one-third the weight of those of heterozygous or wild-type adult littermates (Figure 2). To determine when defective testis development begins in *Dmrt7* mutants, we compared the testes of wild-type and mutant littermates during the first wave of spermatogenesis. Prior to postnatal day 14 (P14), mutant testes appeared histologically normal and the testis weights were similar to those of heterozygous and wild-type littermates, indicating that spermatogonia and early meiotic germ cells form normally (Figure 2B; unpublished data). Thereafter, the testes of the *Dmrt7* mutant mice ceased to grow and the weight difference was significant. Microscopic examination of P21 and P42 *Dmrt7* mutant testes revealed that germ cells arrest in pachynema, and later stages of germ cells are largely missing (Figure 2C and 2D). *Dmrt7* mutant mice are deficient in postmeiotic spermatids and lack epididymal spermatozoa, although a few cells develop to the round spermatid stage. These meiotic defects are in agreement with a recent preliminary analysis of another *Dmrt7* mutation [42]. While some *Dmrt7* mutant tubules are highly vacuolated and contain primarily Sertoli cells and spermatogonia, others have abundant primary spermatocytes. In addition, some tubules contain multinucleated cells and cells with darkly stained nuclei that are typical of apoptotic cells (Figure 2D).

Since *Dmrt7* mutant testes lack most post-pachytene cells, we used TUNEL analysis to test whether the missing cells are eliminated by apoptosis. At 3 wk, *Dmrt7* mutant testes contain significantly more apoptotic cells than those of wild-type controls. The percentage of tubule sections with five or more apoptotic nuclei was about three times higher in *Dmrt7* mutants compared with wild-type (20% versus 7%; Figure 2E). A similar elevation of apoptosis was apparent in mutant testes at 7 wk (Figure 2F). In mutants, many apoptotic cells were in the middle of the tubules, whereas the apoptotic cells in wild-type occur mainly near the seminiferous tubule periphery. The numbers of Sertoli cells were not significantly different between wild-type and mutant testes, and we observed no difference in somatic cell apoptosis in mutants (unpublished data). From these results, we conclude that loss of *Dmrt7* causes a block in meiotic progression, mainly in pachynema, leading to the elimination, by apoptosis, of the arrested cells.

### Pachytene Arrest of *Dmrt7* Mutant Germ Cells

**Figure 3:**
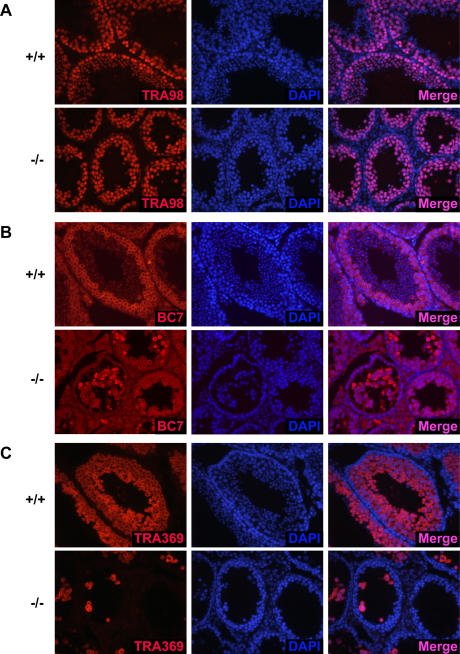
Prophase I Arrest of *Dmrt7* Mutant Germ Cells Testis sections from 6-wk-old wild-type (+/+) and *Dmrt7* mutant (−/−) littermates stained with stage-specific antibodies specific to spermatogenic cells. (A) TRA98 antibody strongly stains spermatogonia, which are present in wild-type and mutant. (B) BC7 antibody stains spermatocytes, which are present in wild-type and mutant. (C) TRA369 antibody stains pachytene and later germ cells, which are severely deficient in the mutant testis.

**Figure 4:**
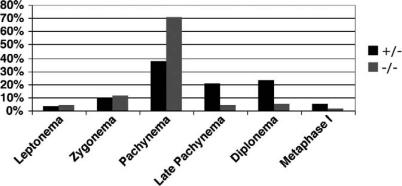
Profile of Meiotic Arrest in *Dmrt7* Mutant Testis Graph of distribution of meiotic stages of germ cells from heterozygous (*n* = 1,970) and homozygous mutant (*n* = 2,084) P26 testes, spread and stained for the synaptonemal complex protein SYCP3 to permit precise staging, which was performed as described [64,65].

To better define the spermatogenic stage at which *Dmrt7^−/−^* male germ cells arrest and die, we used antibodies against several stage-specific germ cell markers. TRA98 is expressed in PGCs and spermatogonia [43]. In the wild-type adult testis, strongly staining TRA98-positive cells form a layer one cell deep; however, in the mutant TRA98, strongly positive cells were abnormally organized, and some tubules had a layer several cells deep (Figure 3A). The BC7 antibody recognizes spermatocytes in the leptotene to early-pachytene stages [44]. *Dmrt7* mutant testes had BC7-positive cells in approximately normal numbers, but again abnormally organized, with many positive cells in the center rather than the periphery of the tubules (Figure 3B). The TRA369 antibody recognizes a calmegin protein expressed in pachytene spermatocytes through elongated spermatids [45]. In contrast to the earlier stages, far fewer TRA369-positive cells were present in mutant testes relative to wild-type (Figure 3C). We also quantitated the number of cells at each meiotic stage using spermatocyte spreads, assaying chromosome-pairing status by staining for SYCP3, a component of the synaptonemal complex (Figure 4). We found that *Dmrt7* mutants accumulate pachytene cells but have greatly reduced numbers of cells in late-pachynema and beyond. Together, these results confirm that the meiotic arrest in *Dmrt7* mutants occurs primarily during pachynema and results in efficient elimination of arrested cells.

### Normal Meiotic Prophase in *Dmrt7* Mutant Germ Cells

**Figure 5:**
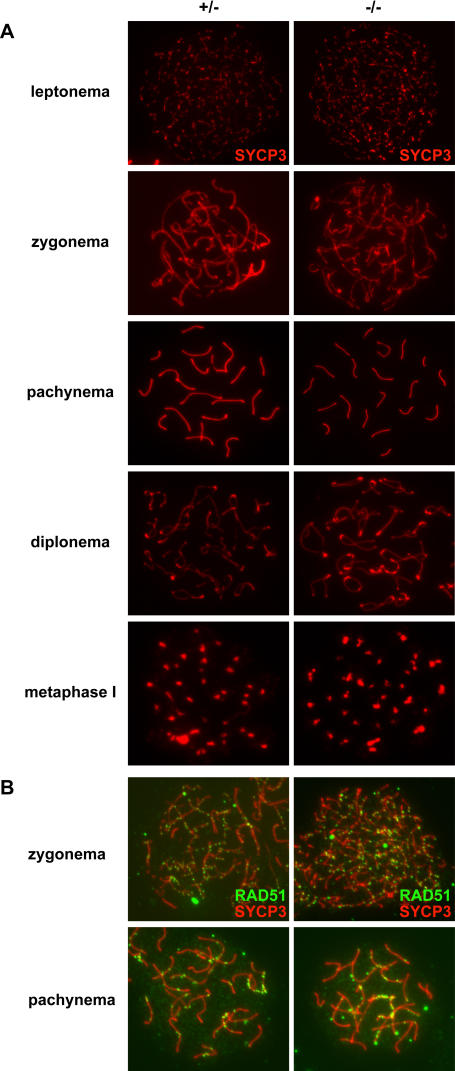
Normal Synapsis and Recombination in *Dmrt7* Mutant Germ Cells (A) Testicular cells from *Dmrt7* heterozygous (+/−) or homozygous (−/−) mutant mice were spread and stained with antibody to SYCP3 (red). The developmental stages of meiotic prophase based on SYCP3 organization are indicated in each panel. By pachynema, all autosomes are fully synapsed, and the XY bivalent is synapsed only at the pseudoautosomal region in both wild-type and mutant cells. (B) Testicular cells in zygonema and pachynema spread and stained with antibodies to RAD51 (green) and SYCP3 (red). Size and distribution of RAD51 foci are similar between wild-type and mutant spermatocytes.

Defects in chromosome pairing, synapsis, or recombination can trigger pachytene arrest and apoptosis [46]. We therefore examined these events in *Dmrt7* mutant testes. To assess homolog synapsis, we used antibodies to SYCP1, a synaptonemal complex transverse element component, and SYCP3, a component of the axial element, which remains on the desynapsed axes during diplonema [47,48]. Formation of synaptonemal complexes in the mutant was indistinguishable from that in wild-type, as indicated by the proper accumulation of SYCP1 (unpublished data) and SYCP3 (Figure 5A). Likewise, the *Dmrt7* mutant zygotene spermatocytes showed normal accumulation of the early recombination repair marker RAD51, suggesting that early meiotic recombination is not significantly affected (Figure 5B). *Dmrt7* mutant spermatocytes exhibited the expected decline in the presence of RAD51 foci associated with the autosomal synaptonemal complexes (Figure 5B; unpublished data) [49]. The few surviving cells that progressed beyond pachynema also underwent apparently normal desynapsis during diplonema (Figure 5A). From these results, we conclude that chromosomal pairing, synapsis, recombination, and desynapsis during prophase I in *Dmrt7* mutant males are grossly normal.

### Abnormal Cellular Organization and the Role of Sertoli Cells

**Figure 6:**
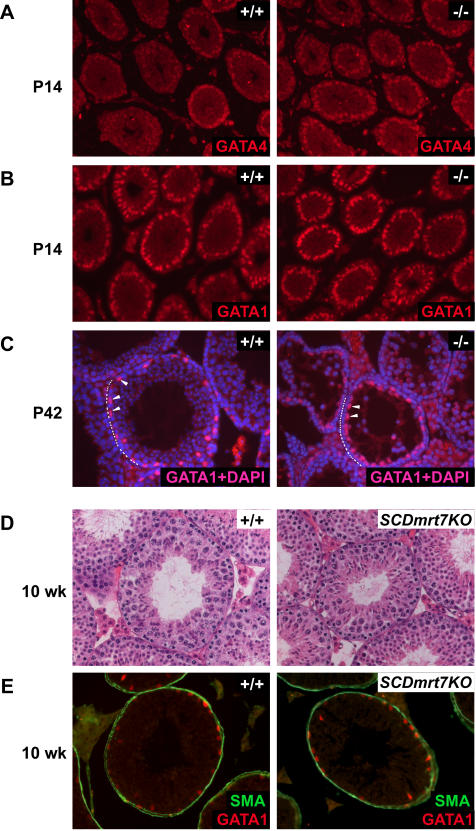
Abnormal Sertoli Cell Organization in *Dmrt7* Mutant Testes (A) Testes from P14 wild-type and *Dmrt7* mutant mice were sectioned and stained with antibody to GATA4. (B) P14 testis sections stained with antibody to GATA1. At P14, GATA4 and GATA1 levels are similar in wild-type and mutant Sertoli cell. (C) Wild-type and mutant testis sections double-stained with antibody to GATA1 (red) and DAPI (blue). Most Sertoli cell nuclei were adjacent to the basal membrane in wild-type, but mutant Sertoli cells were displaced in some tubules (arrowhead). White dotted line indicates position of basal membranes. (D) Testis sections from 10-wk-old wild-type and Sertoli cell-specific *Dmrt7* mutant *(SC-Dmrt7KO)* mice stained with hematoxylin and eosin. Spermatogenesis and spermiogenesis are normal in *SC-Dmrt7KO* testis. (E) Testis sections from 10-wk-old wild-type and *SC-Dmrt7KO* mice stained with antibodies to GATA1 (red) and smooth muscle actin (to outline seminiferous tubules; green). Sertoli cell nuclei are positioned normally near the basal membrane in *SC-Dmrt7KO* mice.

Sertoli cells interact with germ cells during spermatogenesis and the interaction is critical for germ cell maturation [50]. Although we did not detect DMRT7 expression in Sertoli cells by antibody staining, we nevertheless considered the possibility that Sertoli cell defects might contribute to the male-specific germ line failure of *Dmrt7* mutants. To characterize Sertoli cell differentiation, we examined expression of the Sertoli cell markers GATA4 (a marker of immature postnatal Sertoli cells) and GATA1 (a mature Sertoli cell marker). The levels of these proteins appeared normal relative to wild-type at P14 and P42 (Figure 6A–6C), as did the androgen receptor (Figure S3; unpublished data). However, the organization of Sertoli cells in *Dmrt7* mutant testes was abnormal: in some tubules GATA1-positive Sertoli cell nuclei were displaced from their usual close apposition with the basement membrane (Figure 6C). In such tubules, nuclei of pre-meiotic germ cells and spermatocytes were packed close to the basal membrane and few germ cells were found in the adlumenal region.

The aberrant Sertoli cell organization in *Dmrt7* mutant testes raised the possibility that the germ cell phenotype might indirectly result from defects in Sertoli cell function. To test this possibility, we deleted *Dmrt7* just in the Sertoli cell lineage by crossing mice carrying the floxed *Dmrt7* allele with *Dhh-Cre* transgenic mice [51]. The *Desert hedgehog (Dhh)* promoter is active starting at about E12.5 in pre-Sertoli cells but not in germ cells, allowing deletion of *Dmrt7* in Sertoli cells well before any likely requirement for its function [52]. Testicular size in Sertoli-targeted *(SC-Dmrt7KO)* animals was slightly reduced from that of wild-type, but histological analysis revealed no obvious difference between wild-type and *SC-Dmrt7KO* testes (Figure 6D). Spermatogenesis appeared normal, mature sperm were present, and *SC-Dmrt7KO* mice were fertile. In addition, GATA1 staining showed that Sertoli cell nuclei were located adjacent to the basement membrane as in wild-type (Figure 6E). These results suggest the germ cell defects of *Dmrt7* mutants are not caused by lack of *Dmrt7* in Sertoli cells. Rather, the abnormal organization of Sertoli cells appears to result from lack of *Dmrt7* in the germ line.

### XY Bodies Form Normally in *Dmrt7* Mutant Cells

The data presented so far indicate that *Dmrt7* mutant germ cells undergo apparently normal early meiosis and then arrest during pachynema due to a strict requirement for *Dmrt7* in the germ line. To better understand the basis of the meiotic arrest, we more closely examined meiotic germ cells in the mutant. We focused on the XY body, which is thought to be essential for meiotic progression and is the site of preferential DMRT7 localization. Condensation of the X and Y chromosomes begins in late-zygotene cells, and, by mid-pachynema (when homologous chromosome pairs are fully aligned) the sex chromatin forms a microscopically visible spherical structure near the nuclear periphery [53].

**Figure 7:**
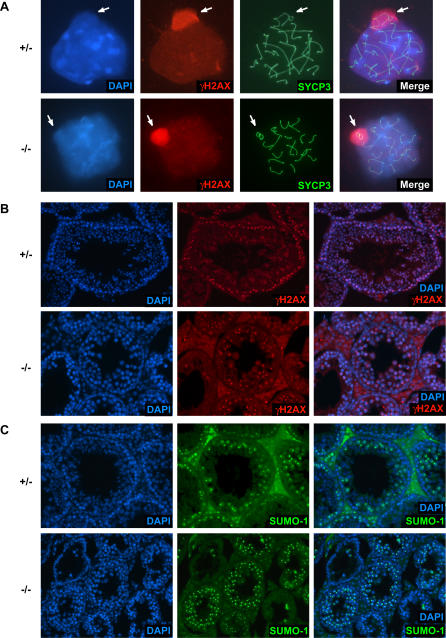
XY Body Forms Normally during Pachynema in *Dmrt7* Mutant Mice (A) Testicular cells spread and stained with antibodies to γH2AX (red) and SYCP3 (green). In pachytene cells, γH2AX is concentrated in the XY body both in wild-type and *Dmrt7* mutant. (B) Testes from 6-wk-old mice sectioned and stained with antibody to γH2AX (red). (C) Testes from 6-wk-old mice sectioned and stained with antibody to SUMO-1 (green). Abundant pachytene cells with XY bodies are present in wild-type and mutant testes.

We first asked whether DMRT7 is required for XY body formation by evaluating several characteristic XY body chromatin features. First, we tested γH2AX expression by immunofluorescent staining. H2AX is a variant of H2A that is crucial for XY body formation and MSCI [12]. γH2AX localized normally to the XY body of DMRT7 mutant cells in meiotic spreads (Figure 7A), and many γH2AX-positive puncta were present in germ cells of *Dmrt7* mutant testes (Figure 7B). Next, we examined SUMO-1 localization in the mutant testis. SUMO-1 expression normally increases in the XY body of early- to mid-pachytene spermatocytes at the time of sex chromosome condensation. Prior to the completion of the first meiotic division, SUMO-1 disappears from the XY body as the X and Y chromosomes desynapse [40]. Punctate SUMO-1 localization was present in *Dmrt7* mutant germ cells, again consistent with formation of a correctly marked XY body (Figure 7C). However, some tubules in mutants had multiple layers of cells with SUMO-1-condensed spots (Figure 7C), rather than the normal single layer of cells. This accumulation of XY body–containing cells also was apparent with γH2AX staining and is consistent with a developmental arrest of mutant cells in mid- to late-pachytene. We also examined Ub-H2A localization in *Dmrt7* mutant testes. In early-pachytene, Ub-H2A is concentrated in the XY body; by mid-pachytene Ub-H2A is observed throughout the entire nucleus, but it again becomes limited to the XY body in late-pachytene spermatocytes [13]. Analysis of nuclear spreads revealed that Ub-H2A localizes normally to the XY body in *Dmrt7* mutants (Figure S4). Collectively, these results indicate that *Dmrt7* mutant germ cells can establish an XY body with at least some of the normal chromatin marks.

### Abnormal Sex Chromatin in *Dmrt7* Mutant Germ Cells

Although the XY body can form during pachynema in *Dmrt7* mutants, we considered the possibility that transcriptional silencing might not be properly established. This would be consistent with the *Dmrt7* phenotype: pachytene cells that escape from MSCI normally are eliminated prior to late-pachytene [17]. Recently, MSCI has been shown to continue into meiosis II and spermiogenesis, apparently mediated by a distinct chromatin compartment termed postmeiotic sex chromatin (PMSC) that is established starting in diplonema [16]. We therefore asked whether the pachytene germ cell death in *Dmrt7* mutants is associated with a failure either to initiate or to maintain sex chromosome inactivation.

**Figure 8:**
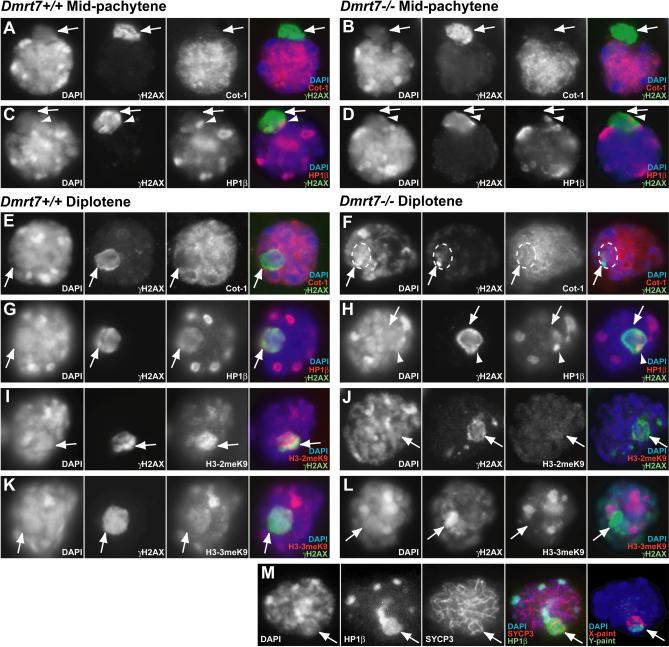
Chromatin Abnormalities in *Dmrt7* Mutant Diplotene Germ Cells (A and B) MSCI occurs normally in mid-pachytene spermatocyte of *Dmrt7* mutant. Arrows indicate XY body in all panels. Cot-1 RNA FISH (red) reveals normal silencing of sex chromosome transcription in XY body. This is consistent with wild-type mid-pachynema as described previously [16]. (C and D) HP1β localization in mid-pachytene spermatocytes. HP1β localizes to X chromosome centromere (arrowhead) in wild-type (C) and mutant (D). (E and F) Cot-1 hybridization in diplotene. Presumptive XY body, based on DAPI and γH2AX localization, is indicated by arrow in (F). (G and H) HP1β in diplotene. Wild-type cell (E) has HP1β throughout XY body, whereas mutant cell (H) only has localization to X chromosome centromere (arrowhead). (I and J) H3-2meK9 localization in diplotene cells. Mutant cell (J) lacks strong concentration of this mark to the XY body seen in wild-type (I). (K and L) H3-3meK9 localization in diplotene cells. Mutant cell (L) has no localization of this mark to the XY body. γH2AX localizes to autosomes in mutant cell, possibly indicating onset of apoptosis. (M) Example of mutant diplotene cell with normal HP1β accumulation to the XY body. All images except those in (M) are single Z sections.

First, we examined the mid-pachytene XY body. To examine XY transcriptional status, we carried out Cot-1 RNA fluorescence in situ hybridization (FISH) to detect nascent RNA polymerase II transcription, combined with DAPI staining to locate the XY body on spreads of seminiferous tubules (Figure 8A and 8B). In *Dmrt7* mutants, the XY body was formed and excluded Cot-1 hybridization (Figure 8B), indicating that transcriptional silencing is established normally in mutant pachytene cells. We also examined expression of the Y-linked gene *Rbmy,* which normally is inactivated during pachytene and reactivated after secondary meiosis begins [54,55]. *Rbmy* was inactivated normally in pachytene cells of *Dmrt7* mutants, based on immunofluorescent staining with an anti-RBMY antibody (Figure S5). We also examined heterochromatin protein 1 beta (HP1β), which normally localizes to the X centromere at mid-pachynema and then spreads through the XY body as it internalizes during diplonema [56]. We found that HP1β localization is normal in DMRT7 mutant cells in mid-pachynema (Figure 8C and 8D). These results suggest that XY body formation and initiation of MSCI both occur normally in *Dmrt7* mutant germ cells.

We next considered the possibility that sex chromatin is established normally but is not properly modified as cells exit pachynema and begin to form PMSC. Although most *Dmrt7* mutant cells are eliminated by apoptosis prior to diplonema, we were able to examine epigenetic markers of PMSC in rare *Dmrt7* mutant spermatocytes that escaped pachytene arrest and progressed into diplonema. First, we examined nascent transcription by Cot-1 hybridization. Although heterochromatic regions generally showed lower Cot-1 signal than euchromatic regions (Figure 8E and 8F), in some mutant cells the sex chromatin appeared to be incompletely silenced relative to wild-type (Figure 8F). We also examined three epigenetic signatures of PMSC: histone H3 dimethylated or trimethylated at lysine-9 (H3-2meK9, H3-3meK9) and spreading of HP1β through the XY body [16,57,58] (S. H. Namekawa, unpublished data). We observed defects in sex chromatin localization of all three markers in *Dmrt7* diplotene cells. Although HP1β localization to the X chromosome centromere initially appeared normal at mid-pachynema, we observed *Dmrt7* mutant diplotene cells that failed to show spreading of HP1β to the entire XY body (Figure 8G and 8H). Similarly, we found *Dmrt7* mutant diplotene cells lacking accumulation of H3-2meK9 and H3-3meK9 marks onto the sex chromatin (Figure 8I–8L).

Not all *Dmrt7* mutant diplotene cells showed abnormal localization of HP1β to the sex chromatin (Figure 8M). In one experiment, 11/27 mutant cells in diplonema lacked HP1β on the XY body, as compared with 2/22 wild-type cells. We hypothesize that the mutant cells with normal HP1β may be those that can complete meiosis (Figures 4 and 5). Some of the mutant diplotene cells showing abnormal sex chromatin also had abnormal autosomal γH2AX staining (Figure 8L). γH2AX localizes to double-strand DNA breaks, so this staining may indicate that some diplotene mutant cells are approaching or entering apoptosis [59]. We did not observe sex chromatin defects prior to diplonema, but we cannot exclude the possibility that earlier defects exist and the affected cells are rapidly eliminated.

**Figure 9:**
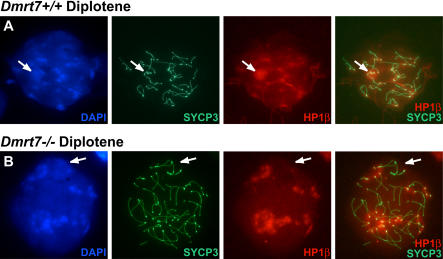
Abnormal Sex Chromatin in Cells Staged by Chromosome Pairing Status (A) Spread of wild-type germ cell stained with DAPI, anti-SYCP3, and anti-HP1β showing chromosome morphology typical of diplonema and internalized XY body with HP1β accumulation. (B) Spread of *Dmrt7* mutant germ cell showing normal diplotene chromosome morphology and internalized XY body, but no HP1β accumulation in the XY body. XY chromosome pairs are indicated by arrow.

In the preceding experiments, we staged cells based on XY body internalization. Because this process could be abnormal in the mutant cells, we also staged mutant cells by chromosome morphology using an antibody to SYCP3 (Figure 9). This independently identified *Dmrt7* mutant diplotene cells lacking HP1β accumulation in the XY body, such as the example in Figure 9B. From these results, we conclude that *Dmrt7* mutant cells establish a normal XY body in mid-pachynema, but then have multiple epigenetic defects in the sex chromatin transition from pachynema to diplonema.

## Discussion

In this study, we find that the DM domain protein DMRT7 is required for male germ cells to complete meiotic prophase but is dispensable in the female germ line. In males, DMRT7 expression is highest in pachytene spermatocytes, and the protein preferentially localizes to the XY body. Consistent with this expression, we found that most mutant male germ cells arrest in pachynema and undergo apoptosis, although a small proportion can progress to diplonema and sometimes beyond. Examination of chromatin and nascent transcription in mutant cells that progressed to diplonema revealed sex chromatin abnormalities, as discussed below.

The pachytene stage of prophase involves tremendous chromosomal changes as the homologs align, synapse, and recombine. During this period, at least one pachytene surveillance system exists to monitor key events of meiotic progression. Cells in which any of these events is anomalous are efficiently eliminated by apoptosis [46]. Another key event of pachynema in male mammals is the packaging of the sex chromosomes into the XY body and the establishment of MSCI. Examination of male meiosis in XYY mice and mice carrying a sex-chromosome-to-autosome translocation showed that cells in which a sex chromosome escapes MSCI are eliminated prior to late-pachynema [17]. This indicates that the establishment of MSCI also is subject to surveillance.

Since the arrest and apoptosis of *Dmrt7* mutant spermatocytes could result from perturbation of any of the critical pachytene events mentioned above, we tested whether they occur abnormally in the mutant cells. We found that chromosomal synapsis and recombination appear normal in *Dmrt7* mutant cells. We therefore focused on the XY body, the most prominent site of DMRT7 accumulation. First, we tested whether the XY body forms and MSCI is established in *Dmrt7* mutant cells. Surprisingly, we found that these cells form an XY body with normal morphology and proper accumulation of all the chromatin marks we examined. Moreover, Cot-1 hybridization and analysis of RBMY expression demonstrated that MSCI initiates normally in the XY body of mid-pachytene *Dmrt7* mutant cells.

We did, however, observe three specific defects in the sex chromatin of *Dmrt7* mutant germ cells that avoided arrest in pachynema and were able to enter diplonema. Normally cells accumulate H3-2meK9 and H3-3meK9 marks and HP1β protein on the sex chromatin as they progress to diplonema, but we observed mutant diplotene cells lacking these features. Thus, although a minority of *Dmrt7* mutant germ cells can progress from pachynema to diplonema, there are defects in sex chromatin modification during the transition. A function in male sex chromatin can reconcile the findings that DMRT7 is required for meiosis, but only in males, and is present only in mammals. A proportion of mutant diplotene cells have apparently normal sex chromatin (for example, Figure 8M); these are likely to be the cells that can progress beyond diplonema.

Because most *Dmrt7* mutant germ cells are eliminated by apoptosis around the time at which we observed sex chromatin defects, a simple model is that the apoptosis is a consequence of the sex chromatin defects. The reciprocal situation (sex chromatin defects caused by apoptosis) is possible, but seems unlikely, because we observed mutant cells with sex chromatin defects but no indications of apoptosis. Alternatively, apoptosis and abnormal sex chromatin may be two independent consequences of *Dmrt7* loss. This question cannot be answered definitively until we know the detailed molecular mechanism of DMRT7.

A number of other proteins have been identified that interact with the XY body, including histone variants and modified histones, a testis-specific histone methyl transferase, chromobox proteins, an orphan receptor germ-cell nuclear factor, and recombination-related proteins [60]. A common feature of these proteins is involvement with heterochromatin and/or transcriptional repression. DMRT7 is unusual among XY body proteins in being related to highly site-specific transcriptional regulators. An attractive speculation is that DMRT7 may provide sequence specificity in recruiting other proteins, such as chromatin modifiers, to the XY body as part of the transition to PMSC. Chromatin regulation may be a common mechanism for DM domain proteins, as we find that other DM domain proteins associate with chromatin modifying complexes (M. W. Murphy, D. Zarkower, and V. J. Bardwell, unpublished data).

The finding that *Dmrt7* is essential for mammalian meiosis expands the known functions of this gene family. Invertebrate DM domain genes so far have only been found to function in somatic cells. Two other DM domain genes, *Dmrt1* and *Dmrt4,* do affect germ cell development in the mouse. *Dmrt1* is required in pre-meiotic male germ cells for differentiation of gonocytes into spermatogonia, as well as in Sertoli cells, but it is not expressed in meiotic cells [31] (S. Kim and D. Zarkower, unpublished data). The requirement for DMRT1 in pre-meiotic germ cells and DMRT7 in meiotic germ cells demonstrates that DM domain proteins act at multiple critical points of male germ cell development. Ovaries of *Dmrt4* mutant females have polyovular follicles (follicles containing multiple oocytes), but it is unknown whether this reflects a defect in the germ line or the soma. It is notable that at least three mammalian DM domain genes affect gonadal development only in one sex, given the similar roles of related proteins in directing sex-specific somatic development in other phyla.

Strikingly, *Dmrt7* is present, not only in placental mammals, but also in marsupials and a monotreme (egg-laying mammal), the platypus, which has a clear *Dmrt7* ortholog [36]. However, no close *Dmrt7* ortholog has been reported in nonmammalian vertebrates, and our database searches did not reveal one. Thus, *Dmrt7* likely arose, presumably by duplication and divergence of another *Dmrt* gene, shortly before or coincident with the mammalian radiation. Monotremes have five X and five Y chromosomes, which form an extended pairing chain during meiosis and appear unrelated to the sex chromosomes of the other mammals [61]. The presence of *Dmrt7* in both lineages may support a common origin for either the sex chromosomes or the sex chromatin of monotremes and other mammals. A plausible model is that *Dmrt7* evolved during the establishment of mammalian sex determination to help cope with ancestral differences in gene dosage, chromosome pairing, recombination, or other meiotic issues arising from sex chromosome heteromorphy. In this regard, we speculate that the recruitment of *Dmrt7* during mammalian evolution may be analogous to the recruitment of chromatin regulatory complexes to achieve somatic dosage compensation during evolution of heteromorphic sex chromosomes in several phyla (reviewed in [62]). It will be of interest to determine whether DMRT7 localizes to sex chromosomes during monotreme meiosis.

In summary, we have found that the mammal-specific DM domain protein DMRT7 is essential for meiotic prophase progression in males. DMRT7 localizes to the sex chromosomes after they are assembled into specialized heterochromatin, and many *Dmrt7* mutant cells have epigenetic defects in the modification of the sex chromatin between pachytene and diplotene. Although *Dmrt7* belongs to an ancient and conserved gene family, it is found only in mammals, and to our knowledge DMRT7 is the only example of a mammal-specific protein that is essential for meiosis. It will be important to determine the precise mechanism by which DMRT7 affects sex chromatin regulation during male meiosis.

## Materials and Methods

### Generation of *Dmrt7* mice.

A mouse *Dmrt7* cDNA fragment containing sequences from exon 8 was used to screen a mouse BAC library from the 129/SvJ strain (Stratagene, http://www.stratagene.com), and clones containing promoter sequences were isolated and sequenced to obtain *Dmrt7* genomic sequence. The targeting vector pDZ169 (diagrammed in Figure S2) was constructed by the following scheme: The vector pDZ157 was used as a backbone vector [31]. 3′ to *Pgk-neo* and the *loxP* site, we inserted, as a XmaI/XmaI DNA fragment, the third intron of *Dmrt7* (from 366 bp to 2,773 bp downstream of exon 3) generated by PCR. 5′ to *Pgk-neo,* we inserted an EcoRI/NotI PCR fragment extending from 4,107 bp to 336 bp 5′ of the *Dmrt7* translational start. Finally, we inserted a *loxP* site and NotI site 336 bp 5′ of the *Dmrt7* translational start. In the resulting vector, the second and third exons of *Dmrt7* are flanked by *loxP* sites (floxed). The *Dmrt7-*containing portions of pDZ169 were completely sequenced.

pDZ169 was linearized with PmeI and electroporated into CJ7 ES cells (originally derived from the 129S1 strain). Three homologous recombinants were identified from 296 G418-resistant colonies by Southern hybridization by use of a DNA probe from the sequences upstream of exon 1 to screen genomic DNA digested with EcoRI. Homologous recombination was confirmed on both ends of the targeted region by Southern hybridization. Probes for Southern hybridization were made by PCR using primers DM5S10/DM5S11 (5′ probe) and DM5PR1/DM5PR2 (3′ probe), listed below. Two targeted ES cell clones containing the floxed allele *Dmrt7^neo^* were injected into C57Bl/6 blastocysts to generate chimeras. Chimeric males were bred with C57Bl/6 females to generate heterozygotes carrying *Dmrt7^neo^. Dmrt7^+^/Dmrt7^neo^* females were bred with male β*-actin-Cre* transgenic mice to delete the floxed sequences and generate heterozygous *Dmrt7^−^* ^/+^ deletion mutants, which were interbred to generate homozygous *Dmrt7^−/−^* mutants.

### Genotyping and RT-PCR.

For genotyping, tail-clip DNA was amplified for 35 cycles. Chromosomal sex was determined by PCR with primers to the Y chromosome gene *Zfy* (below). The wild-type *Dmrt7* allele *Dmrt7^+^* was detected by PCR with DM5S4/DM5S5, with an annealing temperature of 60 °C. The *Dmrt7^flox^* allele was detected by PCR with DM5S5F/DM7KO7R with an annealing temperature of 62 °C. The deleted *Dmrt7* allele *Dmrt7^−^* was detected with DM5S3/DM7KO7R with an annealing temperature of 62 °C. RT-PCR for *Dmrt7* expression analysis was as described [23] using primers SK111/SK112 with an annealing temperature of 62 °C.

### Primers.

DM5S3: 5′-AGAGTGGATTGAATCGGATAGCTC-3′
DM5S4: 5′-AGGATCTTAGTGTCGAATGAATAC-3′
DM5S5: 5′-CCCTTATCCTCCTGCATCCAGATC-3′
DM5S10: 5′-CAGGCTATGGTTAGACTTGAGCAC-3′
DM5S11: 5′-CATCACTCGCGGACAAAGATGCAG-3′
DM5PR1: 5′-CTTCTGCTACAGCCACAGGTCTGG-3′
DM5PR2: 5′-GAATTCAACTAGTATCTGTCCC-3′
DM7KO7R: 5′-CGAGGATCAAGCTCAGGTCACTAGG-3′
DM5S5F: 5′-GATCTGGATGCAGGAGGATAAGGG-3′
ZFYF: 5′-CCTATTGCATGGACAGCAGTCTTATG-3′
ZFYR: 5′-GACTAGACATGTTCTTAACATCTGTCC-3′
SK111: 5′-CCCTTCTGGAAAAGAGAACATAGC-3′
SK112: 5′-GCTCCAGGGGCCTGTGGCTGTAGC-3′
β-*actin*F: 5′-TGCGTGACATCAAAGAGAAG-3′
β-*actin*R: 5′-GATGCCACAGGATTCCATA-3′

### Histological analysis and TUNEL assay.

Dissected testes were fixed in Bouin's fixative or phosphate-buffered formalin overnight at 4 °C, progressively dehydrated in a graded ethanol series, and embedded in paraffin wax. Sections (6 μm) were deparaffinized, rehydrated, and stained with hematoxylin and eosin. For TUNEL analyses, deparaffinized sections were treated with proteinase K for 15 min and quenched in 3.0% hydrogen peroxide in PBS for 5 min at room temperature. Subsequently, nuclear staining in apoptotic cells was detected using ApopTag kit (Chemicon, http://www.chemicon.com) according to the manufacturer's instructions.

### Tissue immunofluorescent staining.

Slides with paraffin sections were washed in PBT (0.1% Tween 20 in PBS) and autoclaved in 10 mM citric acid (pH 6.0) to retrieve antigenicity. Slides were blocked in 5% serum (matched to the species of the secondary antibody) in PBS for 1 h at room temperature and incubated with primary antibodies overnight at 4 °C prior to detection with secondary antibodies.

### Antibodies.

Rabbit polyclonal antibodies to DMRT7 were raised against a purified fusion protein containing glutathione-S-transferase (GST) fused to the C-terminal 279 amino acids of DMRT7. Antibodies to GST were removed by GST-affigel 10 chromatography and the antiserum was then purified by GST-DMRT7-affigel 15 chromatography. DMRT7 antibody was used at 1:200 dilution with a goat anti-rabbit secondary antibody (Molecular Probes, http://www.invitrogen.com) at 1:200 dilution. Other primary antibodies used for immunofluorescence were rat anti-GATA1 (1:200, Santa Cruz Biotechnology, http://www.scbt.com, sc-265), goat anti-GATA4 (1:200, Santa Cruz Biotechnology, sc-1237), rat anti-TRA98 (1:200, gift of H. Tanaka and Y. Nishimune), rat anti-BC7 (1:50, gift of H. Tanaka and Y. Nishimune), rat anti-TRA369 (1:200, gift of H. Tanaka and Y. Nishimune), rabbit anti-RAD51 (1:600 Calbiochem, http://www.calbiochem.com, PC130), mouse anti-GMP-1/SUMO-1 (1:200, Zymed, http://invitrogen.com, 33–2400), rabbit anti-phospho-H2AX (Ser139) (1:200, Upstate, http://www.millipore.com, 01–164), mouse anti-phospho-H2AX (1:200, Upstate, 05–636), mouse anti-SYCP3 (1:200, Abcam, http://www.abcam.com, ab12452), rabbit anti-HP1β (1:100, Abcam, ab10478), rabbit anti-H3-2meK9 (1:100, Upstate, 07–441), rabbit anti-H3-3meK9 (1:200, Upstate, 07–442), rabbit anti-AR (N-20) (1:200, Santa Cruz Biotechnology, sc-816), and mouse anti-αSMA clone 1A4 (1:800, Sigma, http://www.sigmaaldrich.com, A2547). Secondary antibodies used were goat anti-rabbit Alexa 488, goat anti-rabbit Alexa 594, goat anti-rat Alexa 594, and goat anti-mouse Alexa 488 (Molecular Probes) used at 1:250. Donkey anti-goat FITC (Jackson ImmunoResearch Laboratories, http://www.jacksonimmuno.com) and donkey anti-rabbit Texas Red (Jackson) were used at 1:50 according to the manufacturer's instructions.

### Meiotic chromosome spread preparations and FISH.

Meiotic chromosome spread preparations were made from 3-wk-old mice, prepared as described by Reinholdt et al. [63]. For analysis of PMSC and Cot-1 RNA FISH, meiotic slides were prepared as previously described [16]. Slides containing chromosome spreads or meiotic spermatocytes were subjected to immunofluorescent staining or RNA FISH, as previously described [16,63]. For combined RNA FISH/immunostaining, we carried out RNA FISH first, followed by immunofluorescence. DNA FISH was performed using chromosome painting (Cambio, http://www.cambio.co.uk). Z-sections were captured by Zeiss Axioplan microscope (Zeiss, http://www.zeiss.com) and processed by Openlab (Improvision, http://www.improvision.com).

## Supporting Information

Figure S1 Wild-Type and *Dmrt7* Mutant Meiotic Spreads Arrows indicate the location of XY body. SYCP3 antibody staining (green) shows normal synapsis of homologous chromosome synapse in *Dmrt7* mutant cell. DMRT7 protein (red) is localized to the XY body in wild-type (top row) and is not detected in *Dmrt7* mutant (bottom row).
(726 KB JPG)

Figure S2 Targeted Disruption of *Dmrt7*
(A) Diagram of targeting strategy. Homologous recombination of the targeting vector with the wild-type *Dmrt7* allele (*Dmrt7^+^*) resulted in the targeted allele *Dmrt^neo^.* This allele contains *loxP* sites flanking the translational start and the second and third exons (containing the DM domain, gray), as well as a neomycine-resistance cassette flanked by Flp recombinase recognition sites (*frt* sites). Mice heterozygous for the *Dmrt1^neo^* allele were mated with transgenic mice expressing Cre recombinase, resulting in deletion of the sequence between the two *loxP* sites, including the neo cassette. The resulting deletion allele is called *Dmrt7^−^.* Mating of *Dmrt7^neo^* mice with transgenic mice expressing the Flpe recombinase excised the neo cassette, generating the *Dmrt7^flox^* allele.
(B) Southern blots of targeted and wild-type ES cell clones. Genomic DNAs from ES cell clones were digested with NsiI/NotI and EcoRI. The 5′ external probe hybridizes to 14.2-kb (wild-type) and 6-kb (targeted) NsiI/NotI fragments. The 3′ probe hybridizes to 14.6-kb (wild-type) and 4.7-kb (targeted) EcoRI fragments.
(C) Testis sections from adult mice. SUMO-1 (green) on XY body is detected in both wild-type (top) and *Dmrt7* mutant (bottom) section. DMRT7 is detected in wild-type but not detected in *Dmrt7* mutant in SUMO-1-positive cells. (Wild-type images are the same as in Figure 1D.)
(3.4 MB JPG)

Figure S3 Normal Androgen Receptor Expression in *Dmrt7* Mutant Sertoli Cells Testis sections from adult mice stained with antibody to androgen receptor (red) and counterstained with DAPI (blue). In both wild-type (top) and mutant (bottom) testis sections, androgen receptor protein is expressed in Sertoli cell and peritubular myoid cell. Mutant testis section shows abnormal localization of Sertoli cell nuclei, which are displaced from basement membrane.
AR, androgen receptor.
(2.3 MB JPG)

Figure S4 Ub-H2A Localization to XY Body in *Dmrt7* Mutant Spermatocyte Spread pachytene spermatocytes from wild-type (top) and *Dmrt7* mutant (bottom) mice, stained with antibody to Ub-H2A (green) and DAPI (blue). Arrows indicate XY body. Both wild-type and mutant pachytene cells have Ub-H2A properly localized to XY body.
(722 KB JPG)

Figure S5 RBMY Silencing in *Dmrt7* Mutant Spermatocytes (A) SYCP3 (green) and RBMY (red) immunostaining of spread wild-type and *Dmrt7* mutant testicular cells. In wild-type and mutant cells, RBMY is expressed at low levels in leptotene stage. In pachytene spermatocytes, RBMY has fallen to background levels in both wild-type and mutant.
(B) SUMO-1 (green) and RBMY (red) immunostaining of adult testis sections from wild-type and *Dmrt7* mutant. Pre-meiotic cells near the basal membrane express high level of RBMY whereas SUMO-1-positive pachytene cells do not express RBMY, in both wild-type and mutant, indicating proper silencing.
(5.4 MB JPG)

## Abbreviations

DM: Doublesex/MAB-3 domain
ES: embryonic stem
FISH: fluorescence in situ hybridization
GST: glutathione-S-transferase
HP1β: heterochromatin protein 1 beta
γH2AX: phosphorylated H2AX
MSCI: meiotic sex chromosome inactivation
P14: postnatal day 14
PMSC: postmeiotic sex chromatin
RT-PCR: reverse transcriptase-PCR
SUMO-1: small ubiquitin-related modifier 1
Ub-H2A: ubiquitinated H2A
